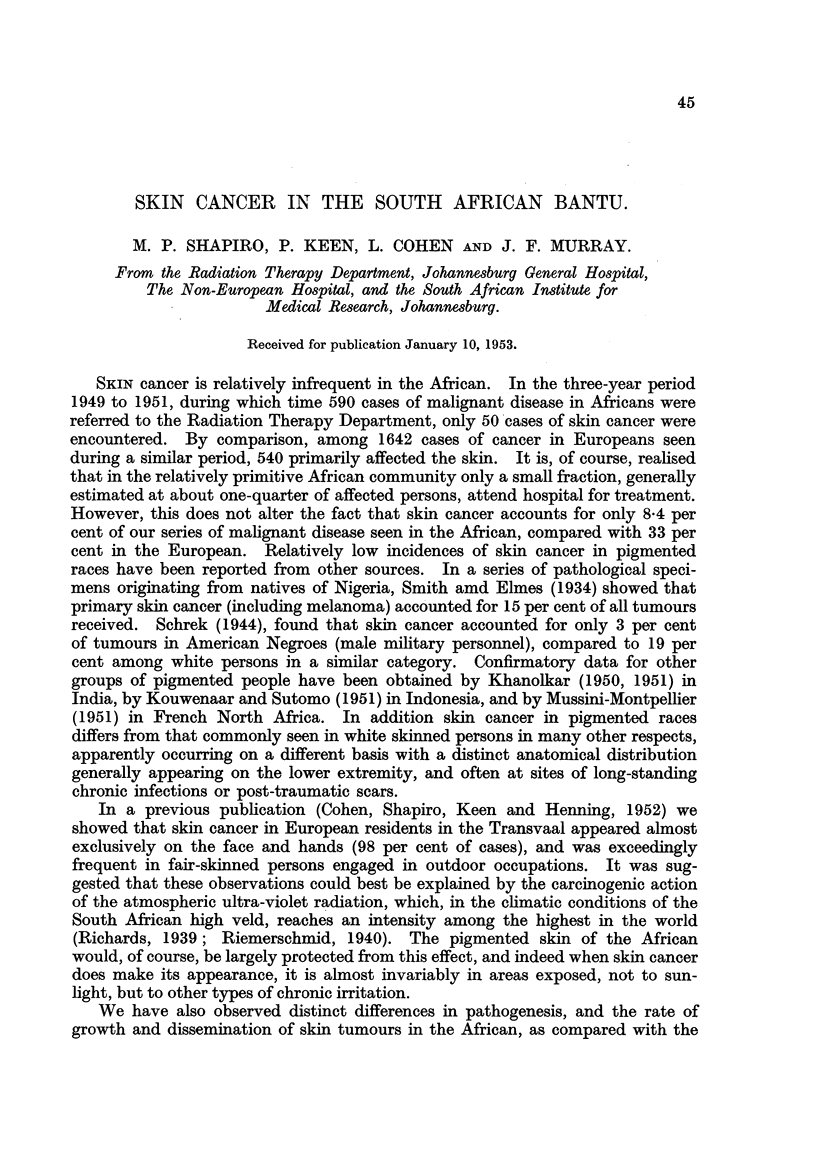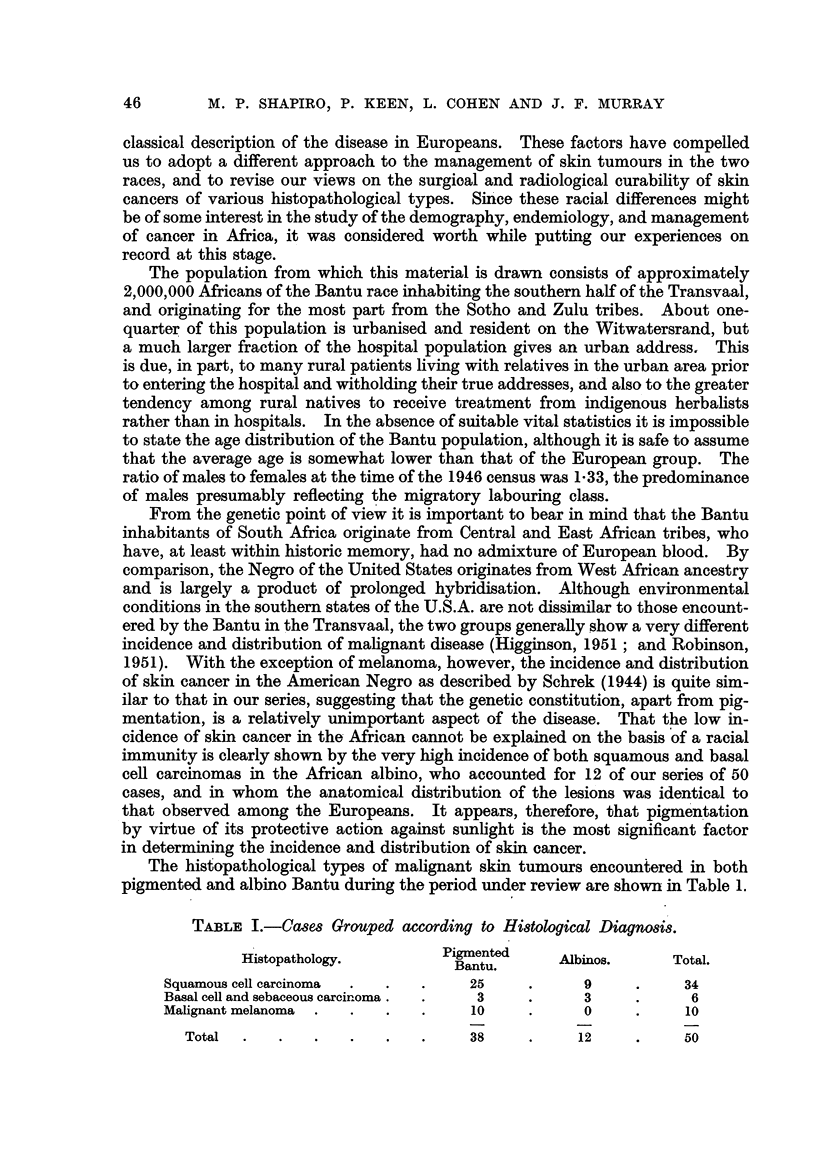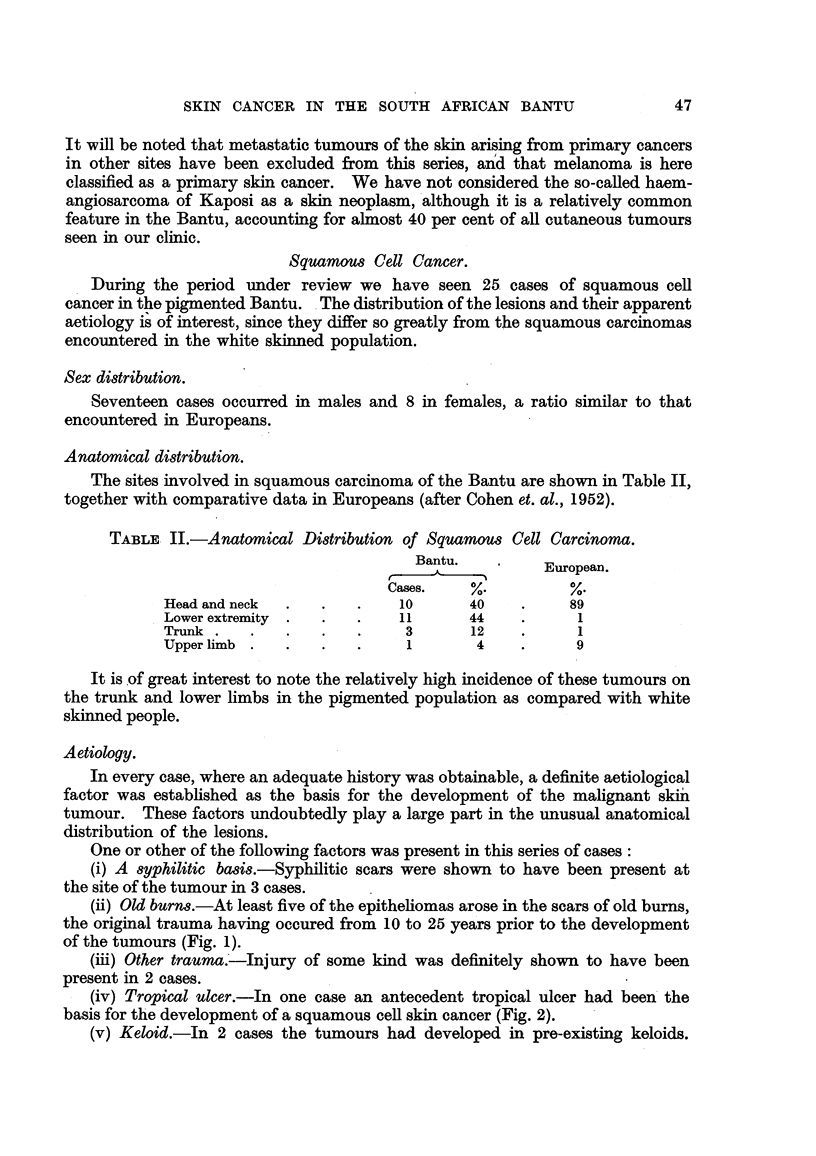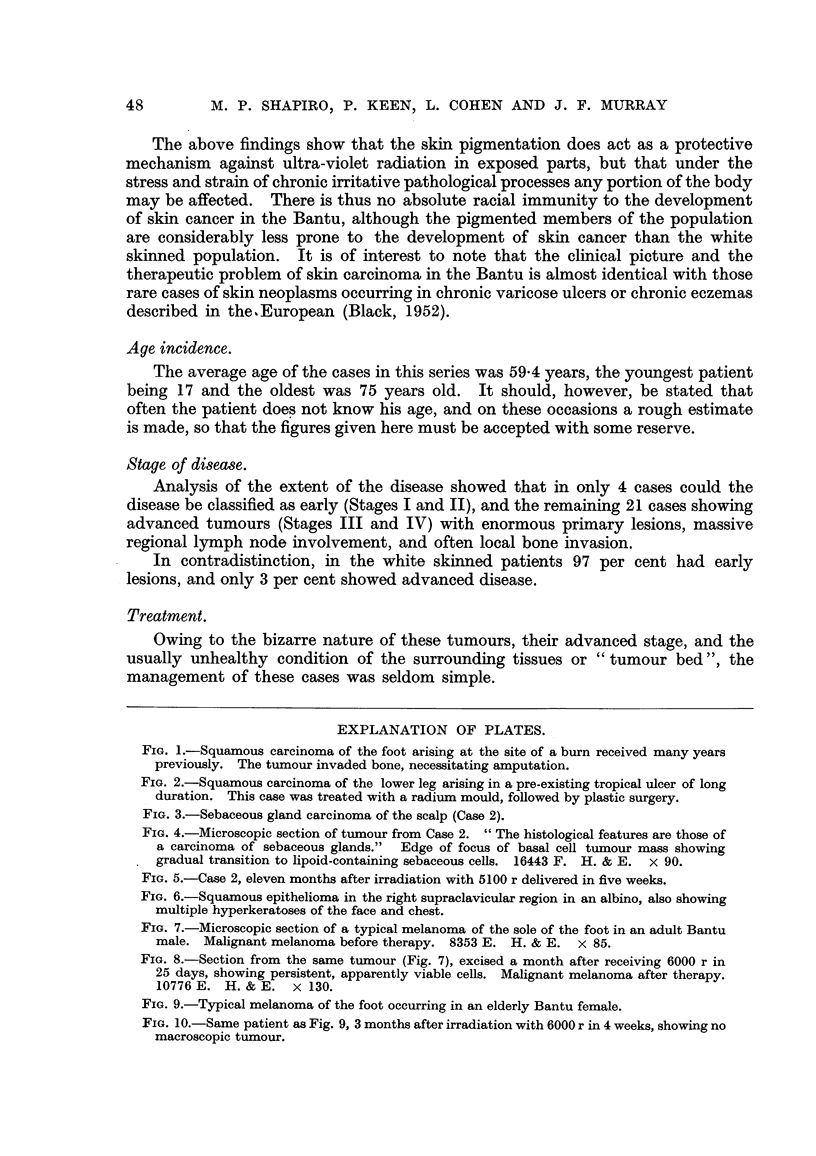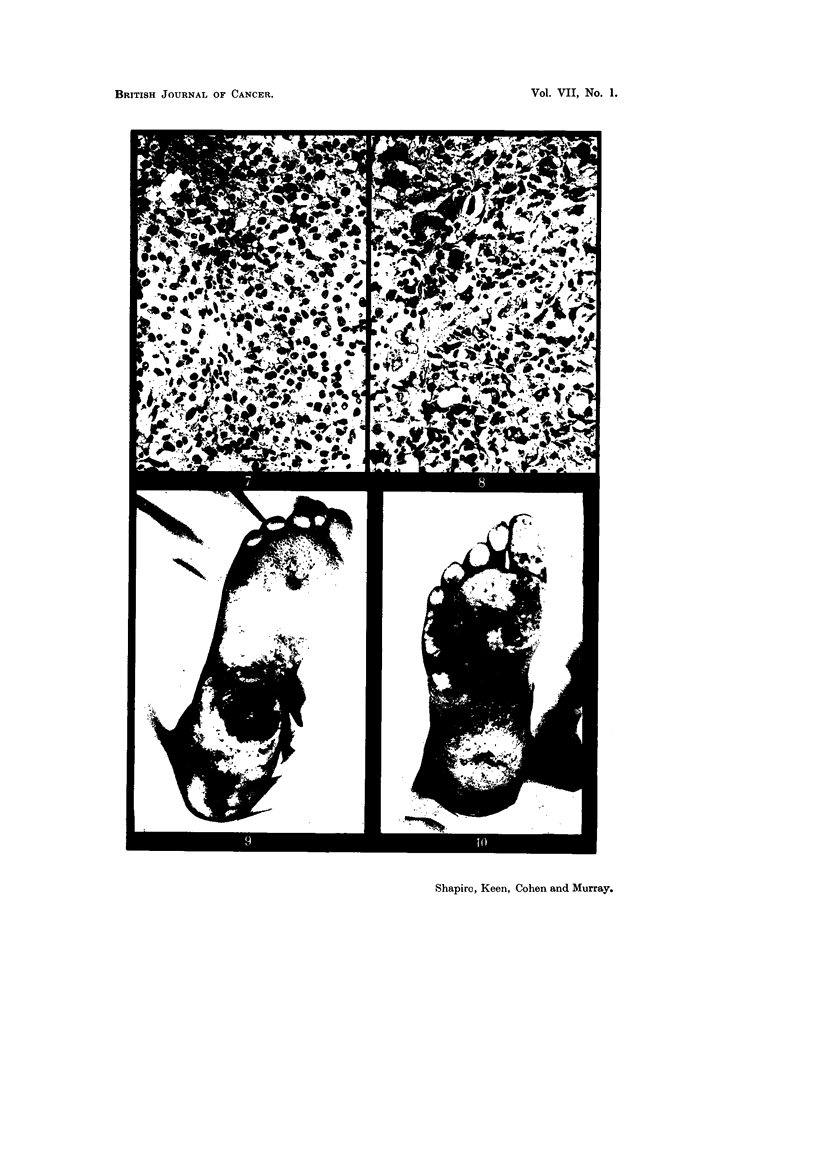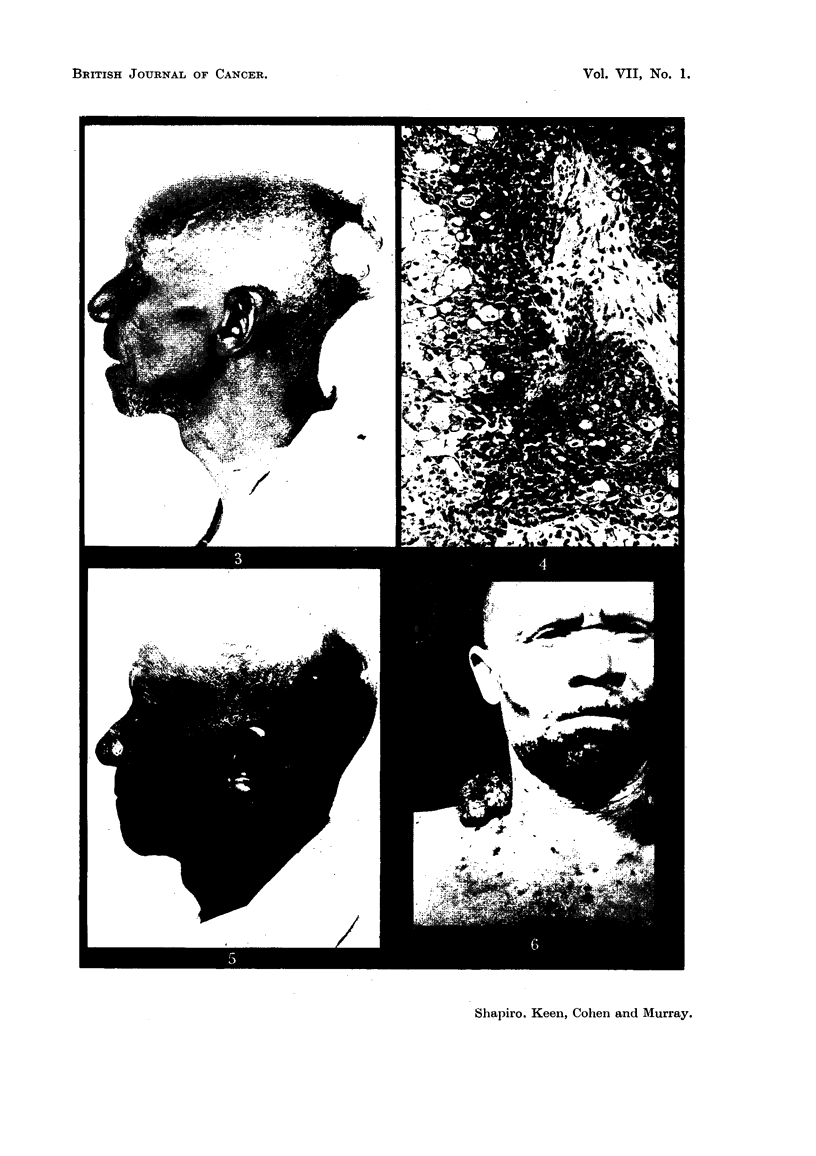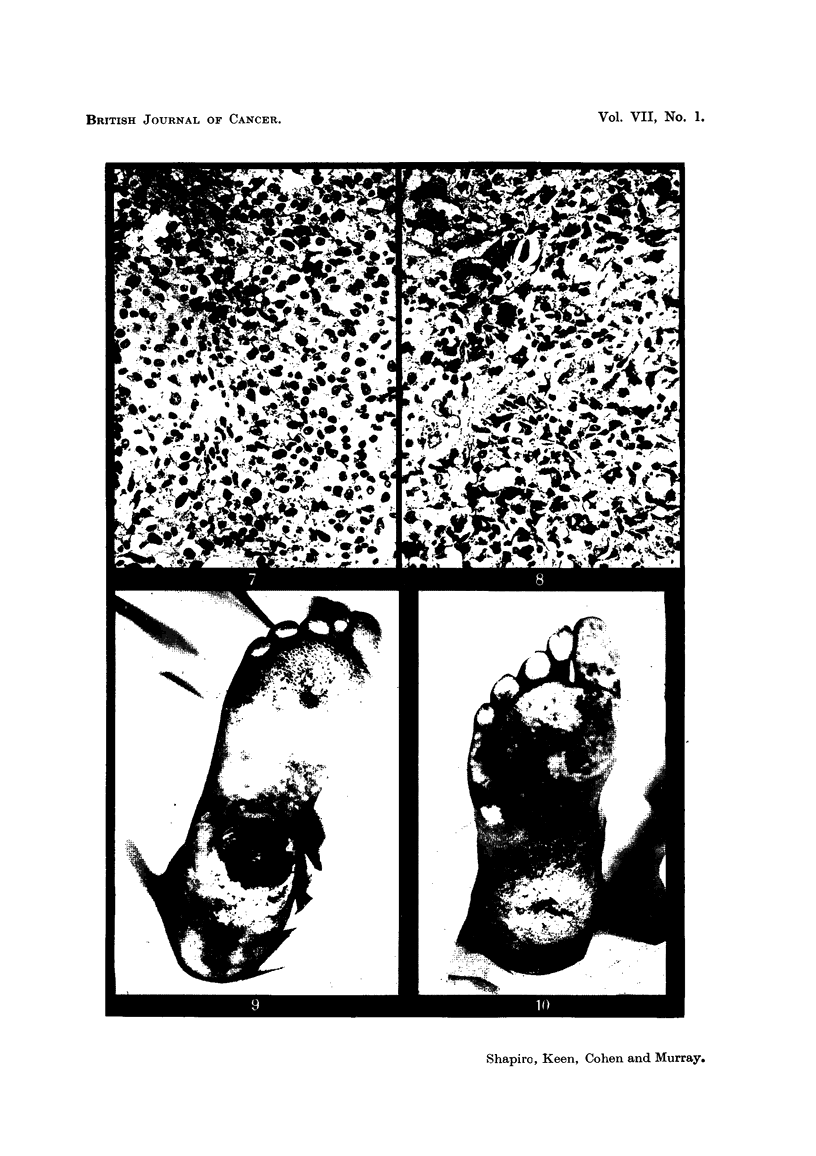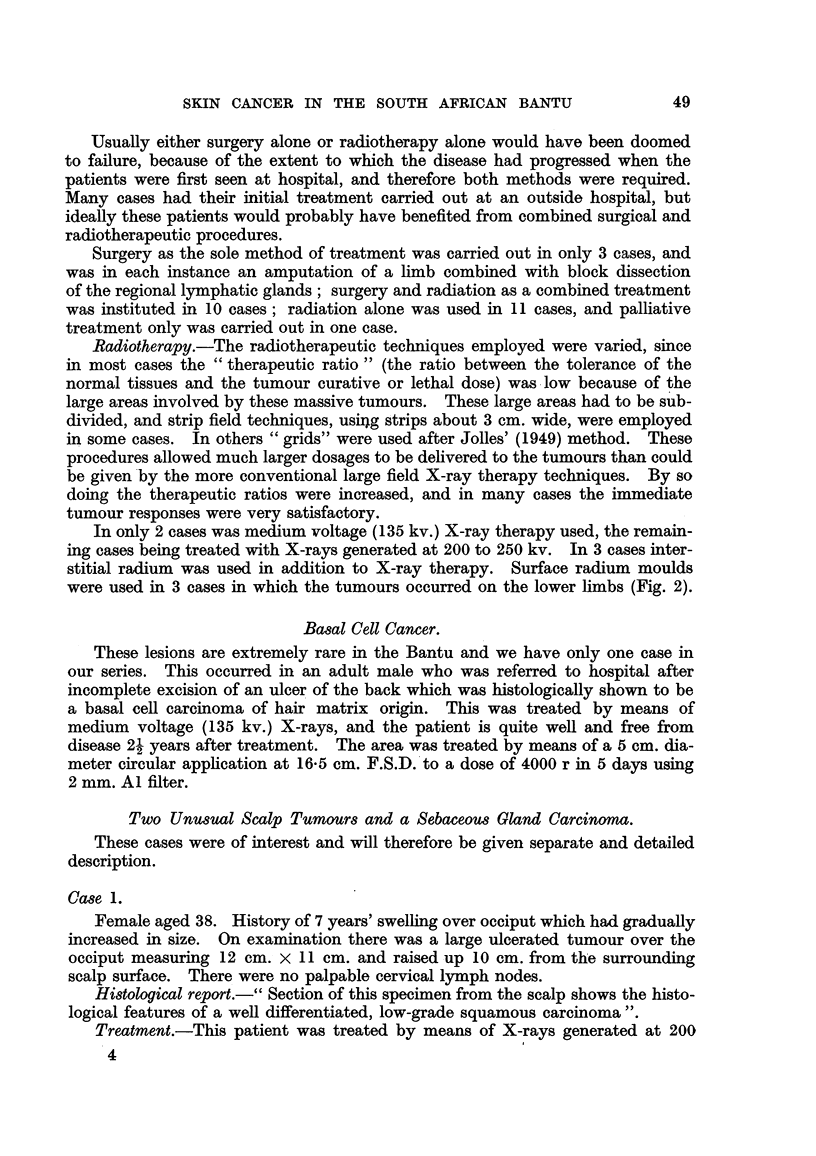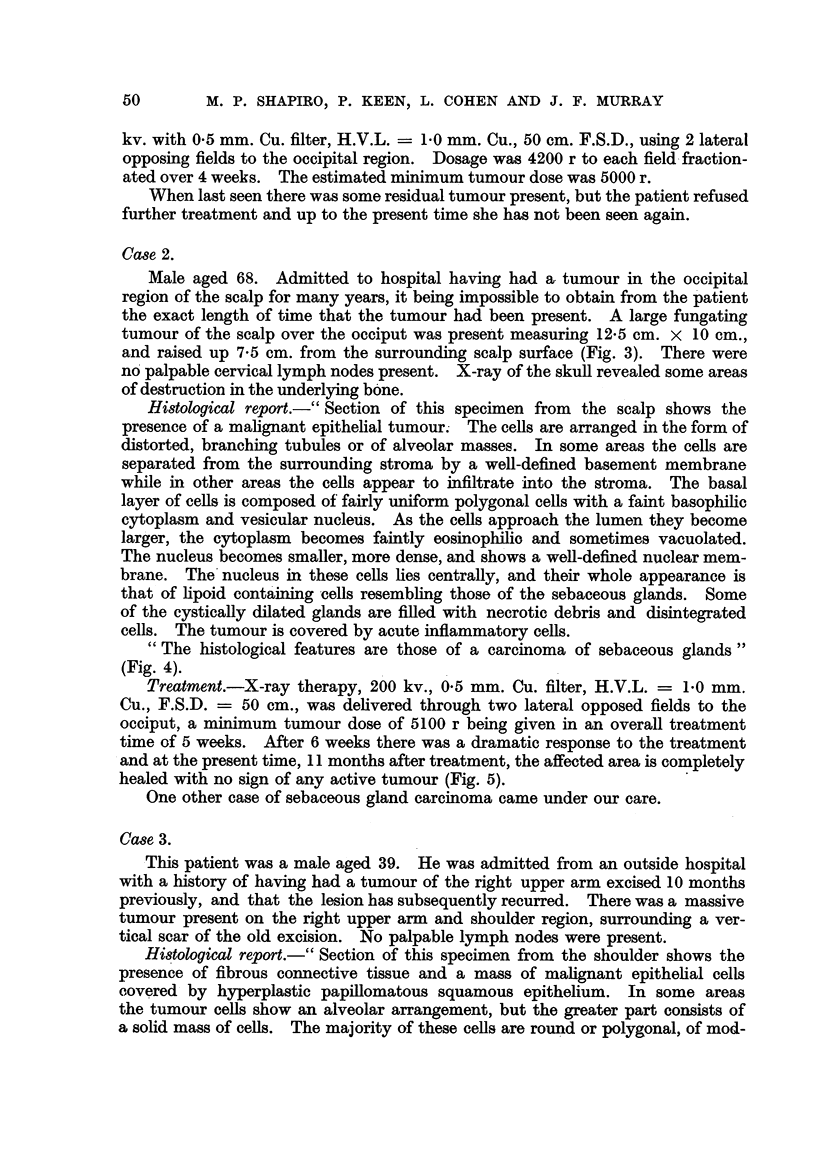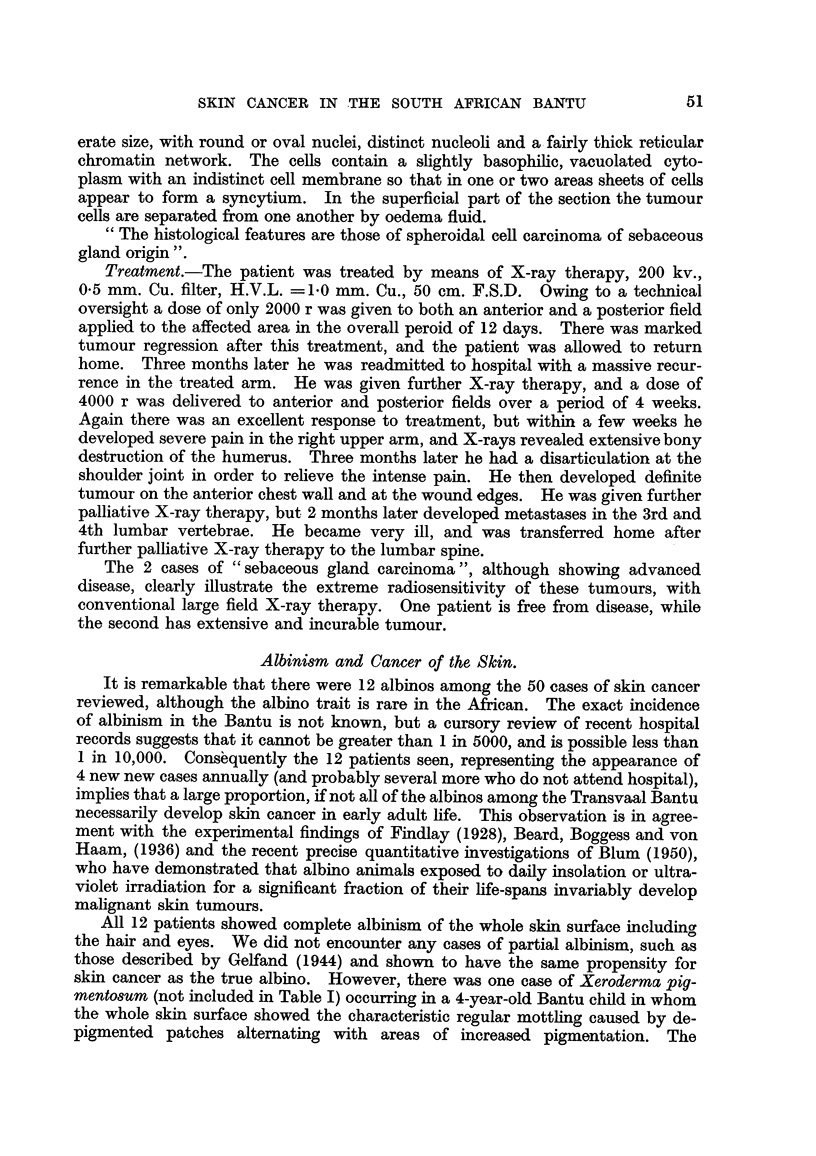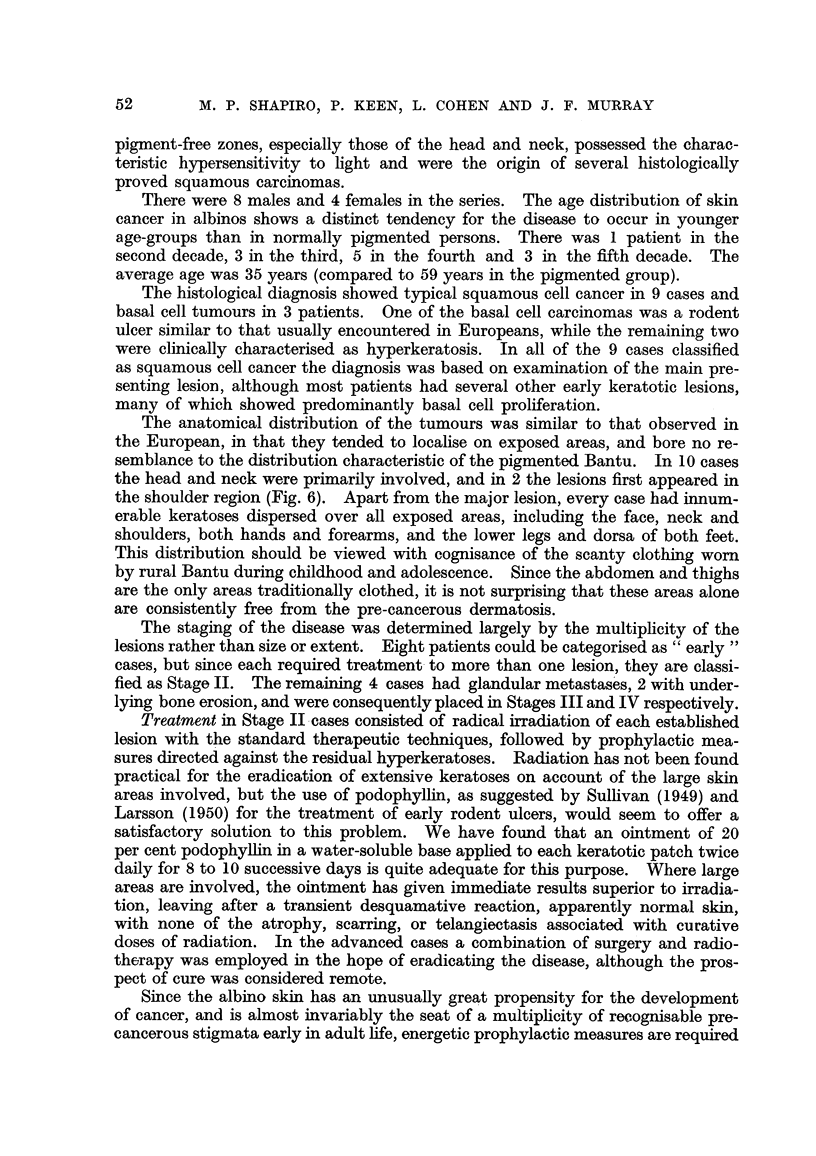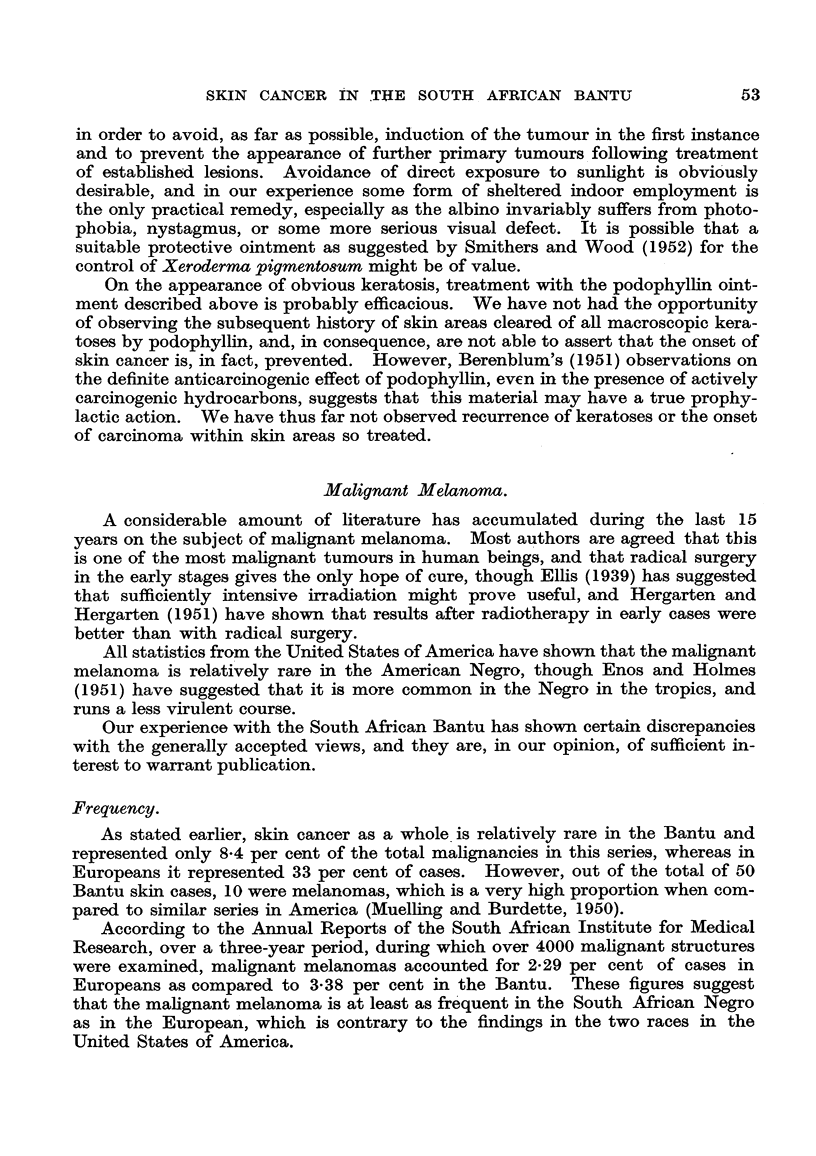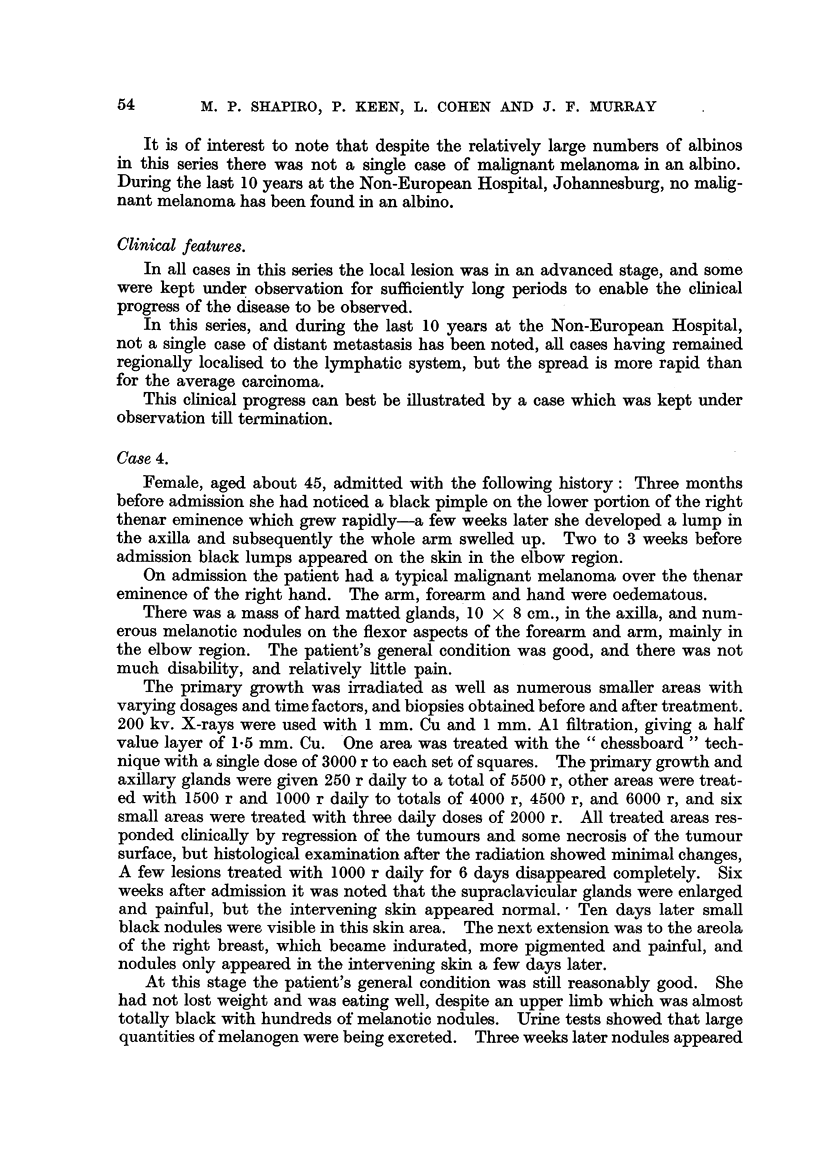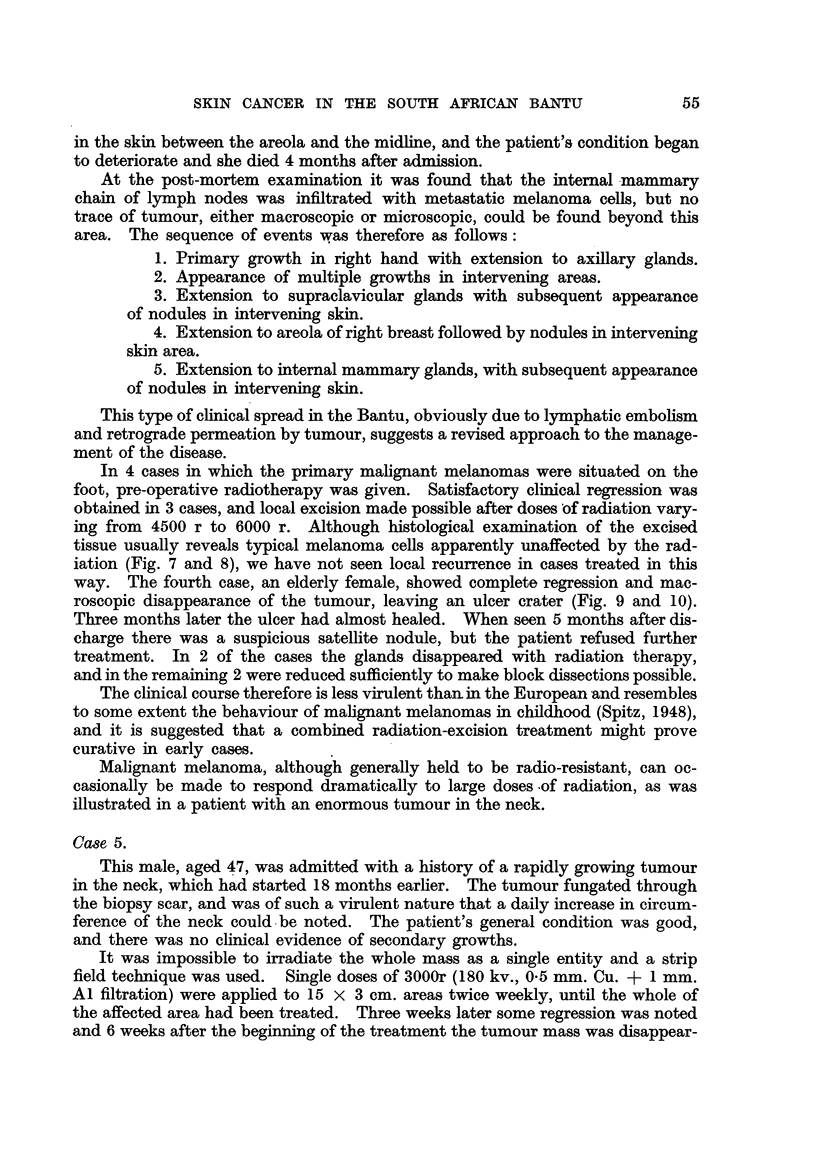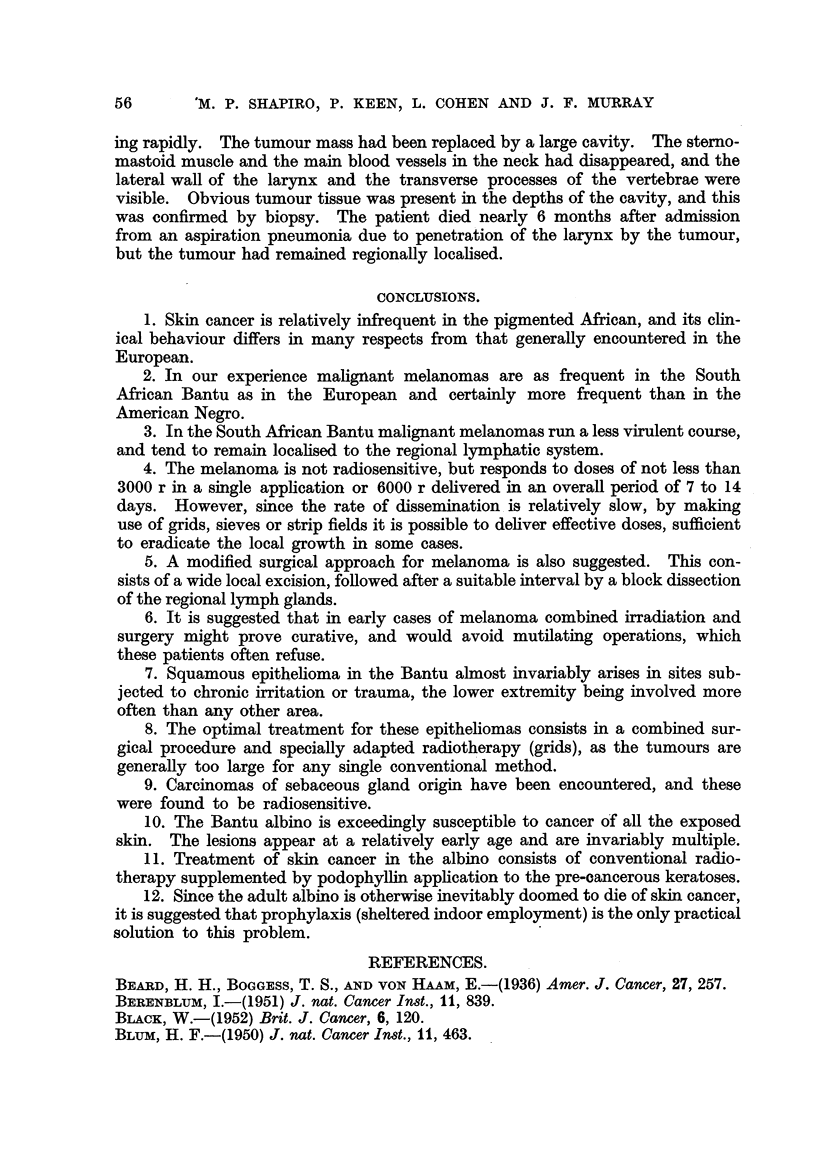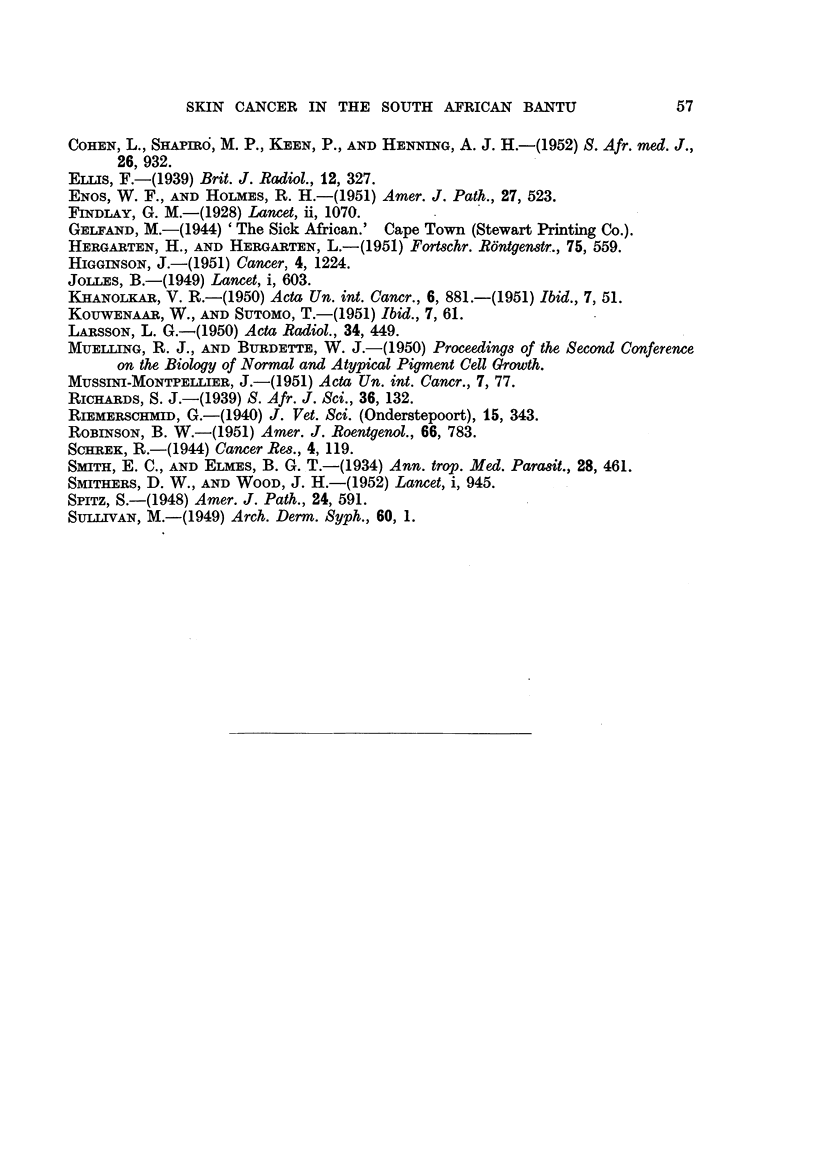# Skin Cancer in the South African Bantu

**DOI:** 10.1038/bjc.1953.5

**Published:** 1953-03

**Authors:** M. P. Shapiro, P. Keen, L. Cohen, J. F. Murray

## Abstract

**Images:**


### SKIN CANCER IN THE SOUTH AFRICAN BANTU.

M. P. SHAPIRO, P. KEEN, L. COHEN AND J. F. MURRAY.

*From the Radiation Therapy Department, Johannesburg General Hospital, The Non-European Hospital, and the South African Institute for Medical Research, Johannesburg.*

Received for publication January 10, 1953.

SKIN cancer is relatively infrequent in the African. In the three-year period 1949 to 1951, during which time 590 cases of malignant disease in Africans were referred to the Radiation Therapy Department, only 50 cases of skin cancer were encountered. By comparison, among 1642 cases of cancer in Europeans seen during a similar period, 540 primarily affected the skin. It is, of course, realised that in the relatively primitive African community only a small fraction, generally estimated at about one-quarter of affected persons, attend hospital for treatment. However, this does not alter the fact that skin cancer accounts for only 8·4 per cent of our series of malignant disease seen in the African, compared with 33 per cent in the European. Relatively low incidences of skin cancer in pigmented races have been reported from other sources. In a series of pathological specimens originating from natives of Nigeria, Smith amd Elmes (1934) showed that primary skin cancer (including melanoma) accounted for 15 per cent of all tumours received. Schrek (1944), found that skin cancer accounted for only 3 per cent of tumours in American Negroes (male military personnel), compared to 19 per cent among white persons in a similar category. Confirmatory data for other groups of pigmented people have been obtained by Khanolkar (1950, 1951) in India, by Kouwenaar and Sutomo (1951) in Indonesia, and by Mussini-Montpellier (1951) in French North Africa. In addition skin cancer in pigmented races differs from that commonly seen in white skinned persons in many other respects, apparently occurring on a different basis with a distinct anatomical distribution generally appearing on the lower extremity, and often at sites of long-standing chronic infections or post-traumatic scars.

In a previous publication (Cohen, Shapiro, Keen and Henning, 1952) we showed that skin cancer in European residents in the Transvaal appeared almost exclusively on the face and hands (98 per cent of cases), and was exceedingly frequent in fair-skinned persons engaged in outdoor occupations. It was suggested that these observations could best be explained by the carcinogenic action of the atmospheric ultra-violet radiation, which, in the climatic conditions of the South African high veld, reaches an intensity among the highest in the world (Richards, 1939; Riemerschmid, 1940). The pigmented skin of the African would, of course, be largely protected from this effect, and indeed when skin cancer does make its appearance, it is almost invariably in areas exposed, not to sunlight, but to other types of chronic irritation.

We have also observed distinct differences in pathogenesis, and the rate of growth and dissemination of skin tumours in the African, as compared with the classical description of the disease in Europeans. These factors have compelled us to adopt a different approach to the management of skin tumours in the two races, and to revise our views on the surgical and radiological curability of skin cancers of various histopathological types. Since these racial differences might be of some interest in the study of the demography, endemiology, and management of cancer in Africa, it was considered worth while putting our experiences on record at this stage.

The population from which this material is drawn consists of approximately 2,000,000 Africans of the Bantu race inhabiting the southern half of the Transvaal, and originating for the most part from the Sotho and Zulu tribes. About one-quarter of this population is urbanised and resident on the Witwatersrand, but a much larger fraction of the hospital population gives an urban address. This is due, in part, to many rural patients living with relatives in the urban area prior to entering the hospital and witholding their true addresses, and also to the greater tendency among rural natives to receive treatment from indigenous herbalists rather than in hospitals. In the absence of suitable vital statistics it is impossible to state the age distribution of the Bantu population, although it is safe to assume that the average age is somewhat lower than that of the European group. The ratio of males to females at the time of the 1946 census was 1·33, the predominance of males presumably reflecting the migratory labouring class.

From the genetic point of view it is important to bear in mind that the Bantu inhabitants of South Africa originate from Central and East African tribes, who have, at least within historic memory, had no admixture of European blood. By comparison, the Negro of the United States originates from West African ancestry and is largely a product of prolonged hybridisation. Although environmental conditions in the southern states of the U.S.A. are not dissimilar to those encountered by the Bantu in the Transvaal, the two groups generally show a very different incidence and distribution of malignant disease (Higginson, 1951 ; and Robinson, 1951). With the exception of melanoma, however, the incidence and distribution of skin cancer in the American Negro as described by Schrek (1944) is quite similar to that in our series, suggesting that the genetic constitution, apart from pigmentation, is a relatively unimportant aspect of the disease. That the low incidence of skin cancer in the African cannot be explained on the basis of a racial immunity is clearly shown by the very high incidence of both squamous and basal cell carcinomas in the African albino, who accounted for 12 of our series of 50 cases, and in whom the anatomical distribution of the lesions was identical to that observed among the Europeans. It appears, therefore, that pigmentation by virtue of its protective action against sunlight is the most significant factor in determining the incidence and distribution of skin cancer.

The histopathological types of malignant skin tumours encountered in both pigmented and albino Bantu during the period under review are shown in Table 1.

TABLE I.—*Cases Grouped according to Histological Diagnosis.*

| Histopathology. | Pigmented Bantu. | Albinos. | Total. |
|---|---|---|---|
| Squamous cell carcinoma | 25 | 9 | 34 |
| Basal cell and sebaceous carcinoma | 3 | 3 | 6 |
| Malignant melanoma | 10 | 0 | 10 |
| Total | 38 | 12 | 50 |

It will be noted that metastatic tumours of the skin arising from primary cancers in other sites have been excluded from this series, and that melanoma is here classified as a primary skin cancer. We have not considered the so-called haemangiosarcoma of Kaposi as a skin neoplasm, although it is a relatively common feature in the Bantu, accounting for almost 40 per cent of all cutaneous tumours seen in our clinic.

*Squamous Cell Cancer.*

During the period under review we have seen 25 cases of squamous cell cancer in the pigmented Bantu. The distribution of the lesions and their apparent aetiology is of interest, since they differ so greatly from the squamous carcinomas encountered in the white skinned population.

*Sex distribution.*

Seventeen cases occurred in males and 8 in females, a ratio similar to that encountered in Europeans.

*Anatomical distribution.*

The sites involved in squamous carcinoma of the Bantu are shown in Table II, together with comparative data in Europeans (after Cohen *et. al.*, 1952).

TABLE II.—*Anatomical Distribution of Squamous Cell Carcinoma.*

| | Bantu. | | European. |
|---|---|---|---|
| | Cases. | %. | %. |
| Head and neck | 10 | 40 | 89 |
| Lower extremity | 11 | 44 | 1 |
| Trunk | 3 | 12 | 1 |
| Upper limb | 1 | 4 | 9 |

It is of great interest to note the relatively high incidence of these tumours on the trunk and lower limbs in the pigmented population as compared with white skinned people.

*Aetiology.*

In every case, where an adequate history was obtainable, a definite aetiological factor was established as the basis for the development of the malignant skin tumour. These factors undoubtedly play a large part in the unusual anatomical distribution of the lesions.

One or other of the following factors was present in this series of cases :

(i) *A syphilitic basis.*—Syphilitic scars were shown to have been present at the site of the tumour in 3 cases.

(ii) *Old burns.*—At least five of the epitheliomas arose in the scars of old burns, the original trauma having occured from 10 to 25 years prior to the development of the tumours (Fig. 1).

(iii) *Other trauma.*—Injury of some kind was definitely shown to have been present in 2 cases.

(iv) *Tropical ulcer.*—In one case an antecedent tropical ulcer had been the basis for the development of a squamous cell skin cancer (Fig. 2).

(v) *Keloid.*—In 2 cases the tumours had developed in pre-existing keloids.

The above findings show that the skin pigmentation does act as a protective mechanism against ultra-violet radiation in exposed parts, but that under the stress and strain of chronic irritative pathological processes any portion of the body may be affected. There is thus no absolute racial immunity to the development of skin cancer in the Bantu, although the pigmented members of the population are considerably less prone to the development of skin cancer than the white skinned population. It is of interest to note that the clinical picture and the therapeutic problem of skin carcinoma in the Bantu is almost identical with those rare cases of skin neoplasms occurring in chronic varicose ulcers or chronic eczemas described in the European (Black, 1952).

*Age incidence.*

The average age of the cases in this series was 59·4 years, the youngest patient being 17 and the oldest was 75 years old. It should, however, be stated that often the patient does not know his age, and on these occasions a rough estimate is made, so that the figures given here must be accepted with some reserve.

*Stage of disease.*

Analysis of the extent of the disease showed that in only 4 cases could the disease be classified as early (Stages I and II), and the remaining 21 cases showing advanced tumours (Stages III and IV) with enormous primary lesions, massive regional lymph node involvement, and often local bone invasion.

In contradistinction, in the white skinned patients 97 per cent had early lesions, and only 3 per cent showed advanced disease.

*Treatment.*

Owing to the bizarre nature of these tumours, their advanced stage, and the usually unhealthy condition of the surrounding tissues or "tumour bed", the management of these cases was seldom simple.

---

EXPLANATION OF PLATES.

FIG. 1.—Squamous carcinoma of the foot arising at the site of a burn received many years previously. The tumour invaded bone, necessitating amputation.

FIG. 2.—Squamous carcinoma of the lower leg arising in a pre-existing tropical ulcer of long duration. This case was treated with a radium mould, followed by plastic surgery.

FIG. 3.—Sebaceous gland carcinoma of the scalp (Case 2).

FIG. 4.—Microscopic section of tumour from Case 2. "The histological features are those of a carcinoma of sebaceous glands." Edge of focus of basal cell tumour mass showing gradual transition to lipoid-containing sebaceous cells. 16443 F. H. & E. × 90.

FIG. 5.—Case 2, eleven months after irradiation with 5100 r delivered in five weeks.

FIG. 6.—Squamous epithelioma in the right supraclavicular region in an albino, also showing multiple hyperkeratoses of the face and chest.

FIG. 7.—Microscopic section of a typical melanoma of the sole of the foot in an adult Bantu male. Malignant melanoma before therapy. 8353 E. H. & E. × 85.

FIG. 8.—Section from the same tumour (Fig. 7), excised a month after receiving 6000 r in 25 days, showing persistent, apparently viable cells. Malignant melanoma after therapy. 10776 E. H. & E. × 130.

FIG. 9.—Typical melanoma of the foot occurring in an elderly Bantu female.

FIG. 10.—Same patient as Fig. 9, 3 months after irradiation with 6000 r in 4 weeks, showing no macroscopic tumour.

7

8

9

10

Shapiro, Keen, Cohen and Murray.

3

4

5

6

Shapiro. Keen, Cohen and Murray.

7

8

9

10

Shapiro, Keen, Cohen and Murray.

Usually either surgery alone or radiotherapy alone would have been doomed to failure, because of the extent to which the disease had progressed when the patients were first seen at hospital, and therefore both methods were required. Many cases had their initial treatment carried out at an outside hospital, but ideally these patients would probably have benefited from combined surgical and radiotherapeutic procedures.

Surgery as the sole method of treatment was carried out in only 3 cases, and was in each instance an amputation of a limb combined with block dissection of the regional lymphatic glands ; surgery and radiation as a combined treatment was instituted in 10 cases ; radiation alone was used in 11 cases, and palliative treatment only was carried out in one case.

*Radiotherapy.*—The radiotherapeutic techniques employed were varied, since in most cases the " therapeutic ratio " (the ratio between the tolerance of the normal tissues and the tumour curative or lethal dose) was low because of the large areas involved by these massive tumours. These large areas had to be subdivided, and strip field techniques, using strips about 3 cm. wide, were employed in some cases. In others " grids" were used after Jolles' (1949) method. These procedures allowed much larger dosages to be delivered to the tumours than could be given by the more conventional large field X-ray therapy techniques. By so doing the therapeutic ratios were increased, and in many cases the immediate tumour responses were very satisfactory.

In only 2 cases was medium voltage (135 kv.) X-ray therapy used, the remaining cases being treated with X-rays generated at 200 to 250 kv. In 3 cases interstitial radium was used in addition to X-ray therapy. Surface radium moulds were used in 3 cases in which the tumours occurred on the lower limbs (Fig. 2).

### *Basal Cell Cancer.*

These lesions are extremely rare in the Bantu and we have only one case in our series. This occurred in an adult male who was referred to hospital after incomplete excision of an ulcer of the back which was histologically shown to be a basal cell carcinoma of hair matrix origin. This was treated by means of medium voltage (135 kv.) X-rays, and the patient is quite well and free from disease $2\frac{1}{2}$ years after treatment. The area was treated by means of a 5 cm. diameter circular application at 16·5 cm. F.S.D. to a dose of 4000 r in 5 days using 2 mm. Al filter.

### *Two Unusual Scalp Tumours and a Sebaceous Gland Carcinoma.*

These cases were of interest and will therefore be given separate and detailed description.

*Case* 1.

Female aged 38. History of 7 years' swelling over occiput which had gradually increased in size. On examination there was a large ulcerated tumour over the occiput measuring 12 cm. × 11 cm. and raised up 10 cm. from the surrounding scalp surface. There were no palpable cervical lymph nodes.

*Histological report.*—" Section of this specimen from the scalp shows the histological features of a well differentiated, low-grade squamous carcinoma ".

*Treatment.*—This patient was treated by means of X-rays generated at 200

kv. with 0·5 mm. Cu. filter, H.V.L. = 1·0 mm. Cu., 50 cm. F.S.D., using 2 lateral opposing fields to the occipital region. Dosage was 4200 r to each field fractionated over 4 weeks. The estimated minimum tumour dose was 5000 r.

When last seen there was some residual tumour present, but the patient refused further treatment and up to the present time she has not been seen again.

*Case* 2.

Male aged 68. Admitted to hospital having had a tumour in the occipital region of the scalp for many years, it being impossible to obtain from the patient the exact length of time that the tumour had been present. A large fungating tumour of the scalp over the occiput was present measuring 12·5 cm. × 10 cm., and raised up 7·5 cm. from the surrounding scalp surface (Fig. 3). There were no palpable cervical lymph nodes present. X-ray of the skull revealed some areas of destruction in the underlying bone.

*Histological report.*—" Section of this specimen from the scalp shows the presence of a malignant epithelial tumour. The cells are arranged in the form of distorted, branching tubules or of alveolar masses. In some areas the cells are separated from the surrounding stroma by a well-defined basement membrane while in other areas the cells appear to infiltrate into the stroma. The basal layer of cells is composed of fairly uniform polygonal cells with a faint basophilic cytoplasm and vesicular nucleus. As the cells approach the lumen they become larger, the cytoplasm becomes faintly eosinophilic and sometimes vacuolated. The nucleus becomes smaller, more dense, and shows a well-defined nuclear membrane. The nucleus in these cells lies centrally, and their whole appearance is that of lipoid containing cells resembling those of the sebaceous glands. Some of the cystically dilated glands are filled with necrotic debris and disintegrated cells. The tumour is covered by acute inflammatory cells.

" The histological features are those of a carcinoma of sebaceous glands " (Fig. 4).

*Treatment.*—X-ray therapy, 200 kv., 0·5 mm. Cu. filter, H.V.L. = 1·0 mm. Cu., F.S.D. = 50 cm., was delivered through two lateral opposed fields to the occiput, a minimum tumour dose of 5100 r being given in an overall treatment time of 5 weeks. After 6 weeks there was a dramatic response to the treatment and at the present time, 11 months after treatment, the affected area is completely healed with no sign of any active tumour (Fig. 5).

One other case of sebaceous gland carcinoma came under our care.

*Case* 3.

This patient was a male aged 39. He was admitted from an outside hospital with a history of having had a tumour of the right upper arm excised 10 months previously, and that the lesion has subsequently recurred. There was a massive tumour present on the right upper arm and shoulder region, surrounding a vertical scar of the old excision. No palpable lymph nodes were present.

*Histological report.*—" Section of this specimen from the shoulder shows the presence of fibrous connective tissue and a mass of malignant epithelial cells covered by hyperplastic papillomatous squamous epithelium. In some areas the tumour cells show an alveolar arrangement, but the greater part consists of a solid mass of cells. The majority of these cells are round or polygonal, of moderate size, with round or oval nuclei, distinct nucleoli and a fairly thick reticular chromatin network. The cells contain a slightly basophilic, vacuolated cytoplasm with an indistinct cell membrane so that in one or two areas sheets of cells appear to form a syncytium. In the superficial part of the section the tumour cells are separated from one another by oedema fluid.

" The histological features are those of spheroidal cell carcinoma of sebaceous gland origin ".

*Treatment.*—The patient was treated by means of X-ray therapy, 200 kv., 0·5 mm. Cu. filter, H.V.L. =1·0 mm. Cu., 50 cm. F.S.D. Owing to a technical oversight a dose of only 2000 r was given to both an anterior and a posterior field applied to the affected area in the overall peroid of 12 days. There was marked tumour regression after this treatment, and the patient was allowed to return home. Three months later he was readmitted to hospital with a massive recurrence in the treated arm. He was given further X-ray therapy, and a dose of 4000 r was delivered to anterior and posterior fields over a period of 4 weeks. Again there was an excellent response to treatment, but within a few weeks he developed severe pain in the right upper arm, and X-rays revealed extensive bony destruction of the humerus. Three months later he had a disarticulation at the shoulder joint in order to relieve the intense pain. He then developed definite tumour on the anterior chest wall and at the wound edges. He was given further palliative X-ray therapy, but 2 months later developed metastases in the 3rd and 4th lumbar vertebrae. He became very ill, and was transferred home after further palliative X-ray therapy to the lumbar spine.

The 2 cases of "sebaceous gland carcinoma", although showing advanced disease, clearly illustrate the extreme radiosensitivity of these tumours, with conventional large field X-ray therapy. One patient is free from disease, while the second has extensive and incurable tumour.

### *Albinism and Cancer of the Skin.*

It is remarkable that there were 12 albinos among the 50 cases of skin cancer reviewed, although the albino trait is rare in the African. The exact incidence of albinism in the Bantu is not known, but a cursory review of recent hospital records suggests that it cannot be greater than 1 in 5000, and is possible less than 1 in 10,000. Consèquently the 12 patients seen, representing the appearance of 4 new new cases annually (and probably several more who do not attend hospital), implies that a large proportion, if not all of the albinos among the Transvaal Bantu necessarily develop skin cancer in early adult life. This observation is in agreement with the experimental findings of Findlay (1928), Beard, Boggess and von Haam, (1936) and the recent precise quantitative investigations of Blum (1950), who have demonstrated that albino animals exposed to daily insolation or ultraviolet irradiation for a significant fraction of their life-spans invariably develop malignant skin tumours.

All 12 patients showed complete albinism of the whole skin surface including the hair and eyes. We did not encounter any cases of partial albinism, such as those described by Gelfand (1944) and shown to have the same propensity for skin cancer as the true albino. However, there was one case of *Xeroderma pigmentosum* (not included in Table I) occurring in a 4-year-old Bantu child in whom the whole skin surface showed the characteristic regular mottling caused by depigmented patches alternating with areas of increased pigmentation. The pigment-free zones, especially those of the head and neck, possessed the characteristic hypersensitivity to light and were the origin of several histologically proved squamous carcinomas.

There were 8 males and 4 females in the series. The age distribution of skin cancer in albinos shows a distinct tendency for the disease to occur in younger age-groups than in normally pigmented persons. There was 1 patient in the second decade, 3 in the third, 5 in the fourth and 3 in the fifth decade. The average age was 35 years (compared to 59 years in the pigmented group).

The histological diagnosis showed typical squamous cell cancer in 9 cases and basal cell tumours in 3 patients. One of the basal cell carcinomas was a rodent ulcer similar to that usually encountered in Europeans, while the remaining two were clinically characterised as hyperkeratosis. In all of the 9 cases classified as squamous cell cancer the diagnosis was based on examination of the main presenting lesion, although most patients had several other early keratotic lesions, many of which showed predominantly basal cell proliferation.

The anatomical distribution of the tumours was similar to that observed in the European, in that they tended to localise on exposed areas, and bore no resemblance to the distribution characteristic of the pigmented Bantu. In 10 cases the head and neck were primarily involved, and in 2 the lesions first appeared in the shoulder region (Fig. 6). Apart from the major lesion, every case had innumerable keratoses dispersed over all exposed areas, including the face, neck and shoulders, both hands and forearms, and the lower legs and dorsa of both feet. This distribution should be viewed with cognisance of the scanty clothing worn by rural Bantu during childhood and adolescence. Since the abdomen and thighs are the only areas traditionally clothed, it is not surprising that these areas alone are consistently free from the pre-cancerous dermatosis.

The staging of the disease was determined largely by the multiplicity of the lesions rather than size or extent. Eight patients could be categorised as " early " cases, but since each required treatment to more than one lesion, they are classified as Stage II. The remaining 4 cases had glandular metastases, 2 with underlying bone erosion, and were consequently placed in Stages III and IV respectively.

*Treatment* in Stage II cases consisted of radical irradiation of each established lesion with the standard therapeutic techniques, followed by prophylactic measures directed against the residual hyperkeratoses. Radiation has not been found practical for the eradication of extensive keratoses on account of the large skin areas involved, but the use of podophyllin, as suggested by Sullivan (1949) and Larsson (1950) for the treatment of early rodent ulcers, would seem to offer a satisfactory solution to this problem. We have found that an ointment of 20 per cent podophyllin in a water-soluble base applied to each keratotic patch twice daily for 8 to 10 successive days is quite adequate for this purpose. Where large areas are involved, the ointment has given immediate results superior to irradiation, leaving after a transient desquamative reaction, apparently normal skin, with none of the atrophy, scarring, or telangiectasis associated with curative doses of radiation. In the advanced cases a combination of surgery and radiotherapy was employed in the hope of eradicating the disease, although the prospect of cure was considered remote.

Since the albino skin has an unusually great propensity for the development of cancer, and is almost invariably the seat of a multiplicity of recognisable precancerous stigmata early in adult life, energetic prophylactic measures are required in order to avoid, as far as possible, induction of the tumour in the first instance and to prevent the appearance of further primary tumours following treatment of established lesions. Avoidance of direct exposure to sunlight is obviously desirable, and in our experience some form of sheltered indoor employment is the only practical remedy, especially as the albino invariably suffers from photophobia, nystagmus, or some more serious visual defect. It is possible that a suitable protective ointment as suggested by Smithers and Wood (1952) for the control of *Xeroderma pigmentosum* might be of value.

On the appearance of obvious keratosis, treatment with the podophyllin ointment described above is probably efficacious. We have not had the opportunity of observing the subsequent history of skin areas cleared of all macroscopic keratoses by podophyllin, and, in consequence, are not able to assert that the onset of skin cancer is, in fact, prevented. However, Berenblum's (1951) observations on the definite anticarcinogenic effect of podophyllin, even in the presence of actively carcinogenic hydrocarbons, suggests that this material may have a true prophylactic action. We have thus far not observed recurrence of keratoses or the onset of carcinoma within skin areas so treated.

### *Malignant Melanoma.*

A considerable amount of literature has accumulated during the last 15 years on the subject of malignant melanoma. Most authors are agreed that this is one of the most malignant tumours in human beings, and that radical surgery in the early stages gives the only hope of cure, though Ellis (1939) has suggested that sufficiently intensive irradiation might prove useful, and Hergarten and Hergarten (1951) have shown that results after radiotherapy in early cases were better than with radical surgery.

All statistics from the United States of America have shown that the malignant melanoma is relatively rare in the American Negro, though Enos and Holmes (1951) have suggested that it is more common in the Negro in the tropics, and runs a less virulent course.

Our experience with the South African Bantu has shown certain discrepancies with the generally accepted views, and they are, in our opinion, of sufficient interest to warrant publication.

### *Frequency.*

As stated earlier, skin cancer as a whole is relatively rare in the Bantu and represented only 8·4 per cent of the total malignancies in this series, whereas in Europeans it represented 33 per cent of cases. However, out of the total of 50 Bantu skin cases, 10 were melanomas, which is a very high proportion when compared to similar series in America (Muelling and Burdette, 1950).

According to the Annual Reports of the South African Institute for Medical Research, over a three-year period, during which over 4000 malignant structures were examined, malignant melanomas accounted for 2·29 per cent of cases in Europeans as compared to 3·38 per cent in the Bantu. These figures suggest that the malignant melanoma is at least as frequent in the South African Negro as in the European, which is contrary to the findings in the two races in the United States of America.

It is of interest to note that despite the relatively large numbers of albinos in this series there was not a single case of malignant melanoma in an albino. During the last 10 years at the Non-European Hospital, Johannesburg, no malignant melanoma has been found in an albino.

*Clinical features.*

In all cases in this series the local lesion was in an advanced stage, and some were kept under observation for sufficiently long periods to enable the clinical progress of the disease to be observed.

In this series, and during the last 10 years at the Non-European Hospital, not a single case of distant metastasis has been noted, all cases having remained regionally localised to the lymphatic system, but the spread is more rapid than for the average carcinoma.

This clinical progress can best be illustrated by a case which was kept under observation till termination.

*Case* 4.

Female, aged about 45, admitted with the following history : Three months before admission she had noticed a black pimple on the lower portion of the right thenar eminence which grew rapidly—a few weeks later she developed a lump in the axilla and subsequently the whole arm swelled up. Two to 3 weeks before admission black lumps appeared on the skin in the elbow region.

On admission the patient had a typical malignant melanoma over the thenar eminence of the right hand. The arm, forearm and hand were oedematous.

There was a mass of hard matted glands, 10 $\times$ 8 cm., in the axilla, and numerous melanotic nodules on the flexor aspects of the forearm and arm, mainly in the elbow region. The patient's general condition was good, and there was not much disability, and relatively little pain.

The primary growth was irradiated as well as numerous smaller areas with varying dosages and time factors, and biopsies obtained before and after treatment. 200 kv. X-rays were used with 1 mm. Cu and 1 mm. Al filtration, giving a half value layer of 1·5 mm. Cu. One area was treated with the " chessboard " technique with a single dose of 3000 r to each set of squares. The primary growth and axillary glands were given 250 r daily to a total of 5500 r, other areas were treated with 1500 r and 1000 r daily to totals of 4000 r, 4500 r, and 6000 r, and six small areas were treated with three daily doses of 2000 r. All treated areas responded clinically by regression of the tumours and some necrosis of the tumour surface, but histological examination after the radiation showed minimal changes, A few lesions treated with 1000 r daily for 6 days disappeared completely. Six weeks after admission it was noted that the supraclavicular glands were enlarged and painful, but the intervening skin appeared normal. Ten days later small black nodules were visible in this skin area. The next extension was to the areola of the right breast, which became indurated, more pigmented and painful, and nodules only appeared in the intervening skin a few days later.

At this stage the patient's general condition was still reasonably good. She had not lost weight and was eating well, despite an upper limb which was almost totally black with hundreds of melanotic nodules. Urine tests showed that large quantities of melanogen were being excreted. Three weeks later nodules appeared in the skin between the areola and the midline, and the patient's condition began to deteriorate and she died 4 months after admission.

At the post-mortem examination it was found that the internal mammary chain of lymph nodes was infiltrated with metastatic melanoma cells, but no trace of tumour, either macroscopic or microscopic, could be found beyond this area. The sequence of events was therefore as follows:

1. Primary growth in right hand with extension to axillary glands.
2. Appearance of multiple growths in intervening areas.
3. Extension to supraclavicular glands with subsequent appearance of nodules in intervening skin.
4. Extension to areola of right breast followed by nodules in intervening skin area.
5. Extension to internal mammary glands, with subsequent appearance of nodules in intervening skin.

This type of clinical spread in the Bantu, obviously due to lymphatic embolism and retrograde permeation by tumour, suggests a revised approach to the management of the disease.

In 4 cases in which the primary malignant melanomas were situated on the foot, pre-operative radiotherapy was given. Satisfactory clinical regression was obtained in 3 cases, and local excision made possible after doses of radiation varying from 4500 r to 6000 r. Although histological examination of the excised tissue usually reveals typical melanoma cells apparently unaffected by the radiation (Fig. 7 and 8), we have not seen local recurrence in cases treated in this way. The fourth case, an elderly female, showed complete regression and macroscopic disappearance of the tumour, leaving an ulcer crater (Fig. 9 and 10). Three months later the ulcer had almost healed. When seen 5 months after discharge there was a suspicious satellite nodule, but the patient refused further treatment. In 2 of the cases the glands disappeared with radiation therapy, and in the remaining 2 were reduced sufficiently to make block dissections possible.

The clinical course therefore is less virulent than in the European and resembles to some extent the behaviour of malignant melanomas in childhood (Spitz, 1948), and it is suggested that a combined radiation-excision treatment might prove curative in early cases.

Malignant melanoma, although generally held to be radio-resistant, can occasionally be made to respond dramatically to large doses of radiation, as was illustrated in a patient with an enormous tumour in the neck.

*Case* 5.

This male, aged 47, was admitted with a history of a rapidly growing tumour in the neck, which had started 18 months earlier. The tumour fungated through the biopsy scar, and was of such a virulent nature that a daily increase in circumference of the neck could be noted. The patient's general condition was good, and there was no clinical evidence of secondary growths.

It was impossible to irradiate the whole mass as a single entity and a strip field technique was used. Single doses of 3000r (180 kv., 0·5 mm. Cu. + 1 mm. Al filtration) were applied to 15 × 3 cm. areas twice weekly, until the whole of the affected area had been treated. Three weeks later some regression was noted and 6 weeks after the beginning of the treatment the tumour mass was disappearing rapidly. The tumour mass had been replaced by a large cavity. The sterno-mastoid muscle and the main blood vessels in the neck had disappeared, and the lateral wall of the larynx and the transverse processes of the vertebrae were visible. Obvious tumour tissue was present in the depths of the cavity, and this was confirmed by biopsy. The patient died nearly 6 months after admission from an aspiration pneumonia due to penetration of the larynx by the tumour, but the tumour had remained regionally localised.

### CONCLUSIONS.

1. Skin cancer is relatively infrequent in the pigmented African, and its clinical behaviour differs in many respects from that generally encountered in the European.
2. In our experience malignant melanomas are as frequent in the South African Bantu as in the European and certainly more frequent than in the American Negro.
3. In the South African Bantu malignant melanomas run a less virulent course, and tend to remain localised to the regional lymphatic system.
4. The melanoma is not radiosensitive, but responds to doses of not less than 3000 r in a single application or 6000 r delivered in an overall period of 7 to 14 days. However, since the rate of dissemination is relatively slow, by making use of grids, sieves or strip fields it is possible to deliver effective doses, sufficient to eradicate the local growth in some cases.
5. A modified surgical approach for melanoma is also suggested. This consists of a wide local excision, followed after a suitable interval by a block dissection of the regional lymph glands.
6. It is suggested that in early cases of melanoma combined irradiation and surgery might prove curative, and would avoid mutilating operations, which these patients often refuse.
7. Squamous epithelioma in the Bantu almost invariably arises in sites subjected to chronic irritation or trauma, the lower extremity being involved more often than any other area.
8. The optimal treatment for these epitheliomas consists in a combined surgical procedure and specially adapted radiotherapy (grids), as the tumours are generally too large for any single conventional method.
9. Carcinomas of sebaceous gland origin have been encountered, and these were found to be radiosensitive.
10. The Bantu albino is exceedingly susceptible to cancer of all the exposed skin. The lesions appear at a relatively early age and are invariably multiple.
11. Treatment of skin cancer in the albino consists of conventional radiotherapy supplemented by podophyllin application to the pre-cancerous keratoses.
12. Since the adult albino is otherwise inevitably doomed to die of skin cancer, it is suggested that prophylaxis (sheltered indoor employment) is the only practical solution to this problem.

### REFERENCES.

Beard, H. H., Boggess, T. S., and von Haam, E.—(1936) *Amer. J. Cancer*, **27**, 257.
Berenblum, I.—(1951) *J. nat. Cancer Inst.*, **11**, 839.
Black, W.—(1952) *Brit. J. Cancer*, **6**, 120.
Blum, H. F.—(1950) *J. nat. Cancer Inst.*, **11**, 463.

Cohen, L., Shapiro, M. P., Keen, P., and Henning, A. J. H.—(1952) *S. Afr. med. J.*, **26**, 932.
Ellis, F.—(1939) *Brit. J. Radiol.*, **12**, 327.
Enos, W. F., and Holmes, R. H.—(1951) *Amer. J. Path.*, **27**, 523.
Findlay, G. M.—(1928) *Lancet*, ii, 1070.
Gelfand, M.—(1944) 'The Sick African.' Cape Town (Stewart Printing Co.).
Hergarten, H., and Hergarten, L.—(1951) *Fortschr. Röntgenstr.*, **75**, 559.
Higginson, J.—(1951) *Cancer*, **4**, 1224.
Jolles, B.—(1949) *Lancet*, i, 603.
Khanolkar, V. R.—(1950) *Acta Un. int. Cancr.*, **6**, 881.—(1951) *Ibid.*, **7**, 51.
Kouwenaar, W., and Sutomo, T.—(1951) *Ibid.*, **7**, 61.
Larsson, L. G.—(1950) *Acta Radiol.*, **34**, 449.
Muelling, R. J., and Burdette, W. J.—(1950) *Proceedings of the Second Conference on the Biology of Normal and Atypical Pigment Cell Growth.*
Mussini-Montpellier, J.—(1951) *Acta Un. int. Cancr.*, **7**, 77.
Richards, S. J.—(1939) *S. Afr. J. Sci.*, **36**, 132.
Riemerschmid, G.—(1940) *J. Vet. Sci.* (Onderstepoort), **15**, 343.
Robinson, B. W.—(1951) *Amer. J. Roentgenol.*, **66**, 783.
Schrek, R.—(1944) *Cancer Res.*, **4**, 119.
Smith, E. C., and Elmes, B. G. T.—(1934) *Ann. trop. Med. Parasit.*, **28**, 461.
Smithers, D. W., and Wood, J. H.—(1952) *Lancet*, i, 945.
Spitz, S.—(1948) *Amer. J. Path.*, **24**, 591.
Sullivan, M.—(1949) *Arch. Derm. Syph.*, **60**, 1.